# A novel family VII esterase with industrial potential from compost metagenomic library

**DOI:** 10.1186/1475-2859-10-41

**Published:** 2011-05-29

**Authors:** Chul-Hyung Kang, Ki-Hoon Oh, Mi-Hwa Lee, Tae-Kwang Oh, Bong Hee Kim, Jung- Hoon Yoon

**Affiliations:** 1 Bioindustry and Bioenergy Research Center, Korea Research Institute of Bioscience and Biotechnology (KRIBB), 125 Gwahangno, Yuseong, Daejeon, Republic of Korea; 2 University of Science & Technology (UST), 217 Gajungro, Yuseoung, Daejeon, Republic of Korea; 3 College of Pharmacy, Chung Nam National University, Daejeon 305-764, Republic of Korea

## Abstract

**Background:**

Among the vast microbial genomic resources now available, most microbes are unculturable in the laboratory. A culture-independent metagenomic approach is a novel technique that circumvents this culture limitation. For the screening of novel lipolytic enzymes, a metagenomic library was constructed from compost, and the clone of *estCS2 *was selected for lipolytic properties on a tributyrin-containing medium.

**Results:**

The *estCS2 *sequence encodes a protein of 570 amino acid residues, with a predicted molecular mass of 63 kDa, and based on amino acid identity it most closely matches (45%) the carboxylesterase from *Haliangium ochraceum *DSM 14365. EstCS2 belong to family VII, according to the lipolytic enzyme classification proposed by Arpigny and Jaeger, and it retains the catalytic triad Ser_245_-Glu_363_-His_466 _that is typical of an α/β hydrolase. The Ser_245 _residue in the catalytic triad of EstCS2 is located in the consensus active site motif GXSXG. The EstCS2 exhibits strong activity toward *p*-nitrophenyl caproate (C6), and it is stable up to 60°C with an optimal enzymatic activity at 55°C. The maximal activity is observed at pH 9, and it remains active between pH 6-10. EstCS2 shows remarkable stability in up to 50% (v/v) dimethyl sulfoxide (DMSO) or dimethylformamide (DMF). The enzyme has the ability to cleave sterically hindered esters of tertiary alcohol, as well as to degrade polyurethanes, which are widely used in various industries.

**Conclusions:**

The high stability of EstCS2 in organic solvents and its activity towards esters of ketoprofen and tertiary alcohols, and in polyurethane suggests that it has potential uses for many applications in biotransformation and bioremediation.

## Background

Discovering microorganisms and their encoded enzymes, and characterizing their interactions, are one of the main purposes in studying microbial diversity. However, it is commonly thought that only 1-10% of the total microorganisms in most environmental samples can be cultured under laboratory conditions. The study of those remaining, known as "unculturables," is important to understand the genetic diversity, population structure, and ecological roles of microbes and to find ways of utilizing them as a novel source of molecules with unique properties [1]. In recent decades, the investigation by function- and sequence- based screening of the entire microbial genome collected directly from a specific environment, the so-called metagenome, has gained much attention as an approach to gather otherwise inaccessible information about microbial communities from various environments as well as to obtain valuable enzymes [2,3].

Composting is a biological decomposition and humification of organic matter by microorganisms. During the thermogenic phase of the composting process, the temperature usually rises up to 80°C for a certain period, and thermophiles belonging to genus *Thermus *or *Bacillus *have been isolated from such material [4,5]. Many enzymes released by microorganisms, such as cellulases, hemicellulases, proteases, phosphatases, arlylsulphatases, and lipases, play key roles in the composting process [6]. Therefore, most of enzymes have thermostable characteristics.

Lipases (EC 3.1.1.3) and esterases (EC 3.1.1.1) are α/β hydrolases that are produced by many microorganisms as well as by eukaryotes. Lipases catalyze the hydrolysis and synthesis of relatively long-chain triacylglycerols, whereas esterases catalyze the reactions involving short-chain triacylglycerols [7,8]. Microbial lipases and esterases classically have been classified into 8 families (Families I-VIII). Recently, additional families were described on the basis of the conserved sequence motifs and biological properties of novel lipolytic enzymes discovered from metagenomic libraries from various environments, such as tidal flat sediment [9,10], surface sea water [11], and deep sea sediment [12,13]. A lipolytic enzyme contains a catalytic triad formed by Ser, His, and Asp/Glu residues, and the Ser residue is usually conserved in the GXSXG pentapeptide motif [14]. Some of the known lipolytic enzymes are stable in various organic solvents, have highly specific chemo-, regional-, and enantioselectivity, and they may be used in the resolution of racemic mixtures and synthesis of pharmaceuticals and new surfactants by bioconversion of oils, fats, etc. [7,15]. Interestingly, some esterases degrade polyurethane, which is a hydrophobic synthetic polymer. For example, *Comamonas acidovorans *TB-35 and *Pseudomonas chlororaphis *encode esterases that can degrade this polymer [16,17].

In this study, we report the screening of a metagenome library, constructed from compost, and the isolation of a unique thermostable lipolytic enzyme. We identified the esterase as belonging to family VII, and we describe the biochemical characterization of purified protein.

## Results

### Screening of esterase clones and DNA sequence analysis

Screening a metagenomic fosmid library for esterase-positive clones was conducted by identifying the areas of halo formation around bacterial colony margins on the tributyrin agar plates. Ten of the positive clones were identified among approximately 13,000 chloramphenicol-resistant recombinant clones, and one of these positive clones was selected for further study.

In order to find the gene encoding lipolytic activity within the fosmid, subcloning of randomly digested DNA fragments, generated by partial *Sau*3AI digestion of the fosmid, and activity screening of the subclones, were performed. One open reading frame, designated *estCS2*, was identified. DNA sequence analysis revealed that *estCS2 *consists of 1,713 bp that encode a protein of 530 amino acids, with a predicted molecular weight of 63 kDa.

**Figure 1:**
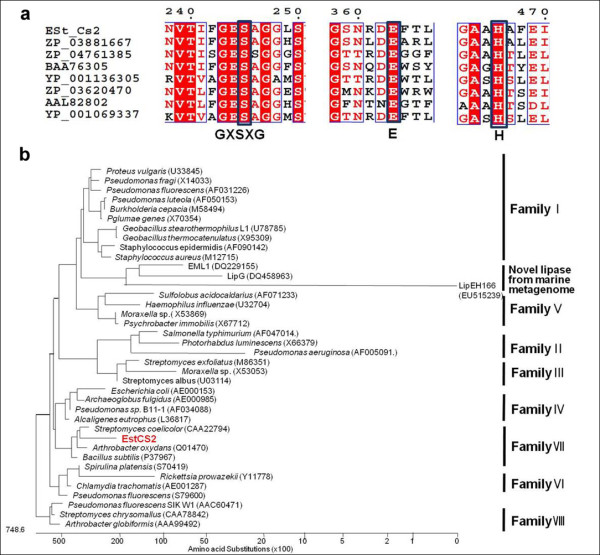
**Multiple amino acid sequence alignments and phylogenetic analysis of EstCS2.** (a) Multiple amino acid sequence alignments of EstCS2 and its homologs. ZP_03881667, carboxylesterase from *Haliangium ochraceum *DSM 14365; ZP_04761385, Carboxylesterase from *Acidovorax delafieldii *2AN; BAA76305, polyurethane esterase from *Delftia acidovorans *YP_001136305, carboxylesterase from *Mycobacterium gilvum *PYR-GCK; ZP_03620470, Carboxylesterase from *Tolumonas auensis *DSM 9187 AAL82802, paraben-hydrolyzing esterase precursor from *Enterobacter cloacae*; YP_001069337, carboxylesterase, from *Mycobacterium *sp. JLS. (b) Phylogenetic tree based on amino acid sequence of EstCS2 and closely related proteins. Phylogenetic analysis was performed using the program MEGALIGN (DNASTAR, Madison, WI). Except EstCS2, protein sequences for previously identified families of bacterial lipolytic enzymes were retrieved from GenBank (http://www.ncbi.nlm.nih.gov). The units at the bottom of the tree indicate the number of substitution events.

The amino acid sequence of EstCS2 exhibits 45% identity and 58% similarity to a carboxylesterase from *Haliangium ochraceum *DSM 14365 (Genbank:ACY17267), 40% identity and 52% similarity to a carboxylesterase from *Acidovorax delafieldii *2AN (Genbank:EER61780.1), and 39% identity and 52% similarity to a polyurethane esterase from *Delftia acidovorans *(Genbank:BAA76305). The catalytic triad of EstCS2 was identified as Glu_363_-His_466_-Ser_375 _(Ser_375 _is contained within the classical GXSXG pentapeptide motif at amino acid positions 373-377), through multiple sequence alignment with closely related homologues [14,18]. Phylogenetic analysis of EstCS2 and other lipases reveals that EstCS2 belongs to family VII (Figure 1)[13].

### Cloning of *estCS2*, and overexpression and purification of the enzyme

*E.coli *BL21(DE3) was transformed using a recombinant plasmid constructed from pET-estCS2 and inserted *estCS2 *sequences (pET-estCS2). Following induction, the encoded EstCS2 was expressed in an active form in the soluble fraction of the host cells. By purification on nickel-nitrilotriacetic acid (Ni-NTA) resin, EstCS2 was purified at a 41% yield from the soluble fraction, and the specific activity was increased to approximately 6-fold. By sodium dodecyl sulfate-polyacrylamide gel electrophoresis (SDS-PAGE) analysis under denaturing conditions, the enzyme has an apparent molecular mass of 55 kDa. The N-terminal sequence of EstCS2 was determined to identify them. N-terminal amino acid sequencing of purified EstCS2 allowed the identification of 5 amino acid residues, D_60_DREP_65_. The lipolytic activity of the protein was confirmed by a zymogram on an indicator plate, as a clear band corresponding to a size of 55 kDa.

### Characterization of esterase EstCS2

**Figure 2:**
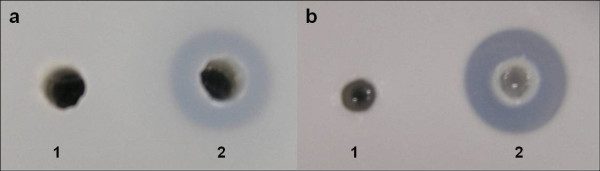
**Plate assay for degradation of polyurethane and triglyceride by EstCS2**. (a) polyurethane degrading activity plate, (b) lipolytic activity plate, 1. Crude cell extract of *E.coli *BL21(DE3)/pET22b, 2. Purified EstCS2.

Because the sequence of EstCS2 is closely related to the polyurethane esterase from *Deftia acidvorans *TB-35, polyurethane-degrading activity of EstCS2 was determined on an indicator plate containing poly(diethylene glycol adipate)(poly DEGA). The purified EstCS2 formed a clear zone on the indicator plate, indicating the enzyme can hydrolyze poly DEGA. EstCS2 also formed a clear zone on a lipolytic activity indicator plate containing tributyrin as the substrate (Figure 2).

**Figure 3:**
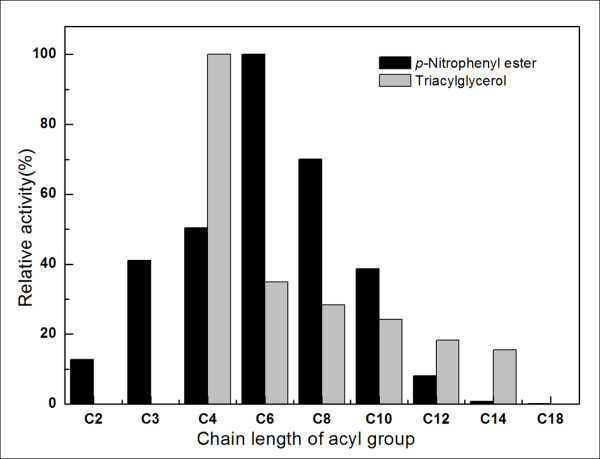
**Substrate specificity of EstCS2 toward various triacylglycerols and *p*-nitrophenyl esters**.

Enzyme specificity with respect to the length of the acyl chains in the substrate was investigated using *p-*nitrophenyl esters as substrates. The esterase showed highest activity with *p-*nitrophenyl caproate (pNPC, C6) among the *p-*nitrophenyl esters tested, while *p*-nitrophenyl esters with long-chain acyl groups were not hydrolyzed efficiently. Results of measuring free fatty acids released enzymatically, which was monitored by change in pH, also showed that EstCS2 has high lipolytic activity toward short chains, especially tributyrin (C4) (Figure 3).

**Figure 4:**
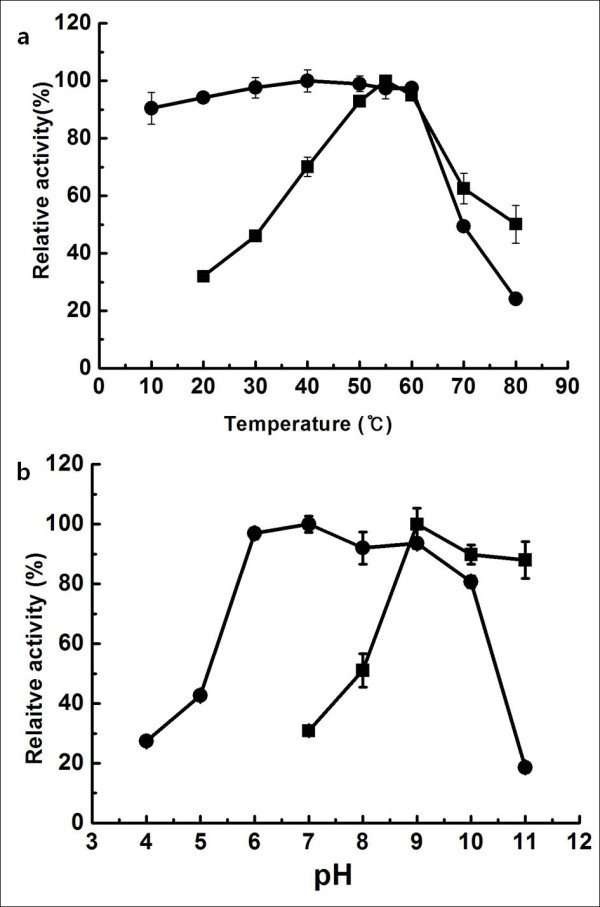
**Effect of temperature and pH on the activity of EstCS2**. (a) Enzyme activity was measured at each temperature under standard assay condition (■). In addition, the enzyme was preincubated at the indicated temperature, and the remaining activity was determined (●). (b) Enzyme activity was measured at each pH under standard assay conditions (■). In addition, the enzyme was preincubated at the indicated pH, and the remaining activity was determined (●).

Effect of temperature on the activity of EstCS2 was investigated over a range of 10-80°C, and these experiments indicated that EstCS2 has an optimal activity at 55°C and exhibits 50% of the maximal activity at 80°C. Thermostability data showed that EstCS2 is stable up to 60°C, but this stability decreases sharply at temperatures above 70°C (Figure 4a). This activity at high temperatures is a common feature of thermostable enzymes [19]. In order to determine the optimal pH for the esterase, we measured the activity of EstCS2 at various pH values (pH 7-11), using pNPC as a substrate, at 25°C. EstCS2 exhibited the highest activity at pH 9.0, and the esterase was found to be stable within a wide pH range (pH 6.0-10.0) (Figure 4b).

### Effect of additives

Various detergents, such as 0.1% (w/v) Tween 20, Tween 40, Tween 80, Triton X-100, Na-taurocholate, Na-deoxycholate, and CHAPS, slightly increased enzyme activity, while 1% (w/v) SDS reduced the activity by about 12%. The effects of divalent metal ions on EstCS2 activity were tested, which revealed that 1 mM CaCl_2 _slightly increases the activity (119%), and 10 mM FeSO_4 _is inhibitory.

To investigate the amino acid residues involved in catalysis, the inhibitory effect of various chemical modifiers was examined. The activity of EstCS2 was inhibited by phenylmethylsulfonyl fluoride (PMSF), as are other lipolytic enzymes that contain a serine residue in the active site in a conserved pentapeptide GXSXG [20].

### The effect of organic solvents on the activity of EstCS2

To test the effect of solvents on esterase activity, various solvents were added to the enzyme reaction, at final concentrations of 10, 20, and 50% (v/v) of the total volume. In 10% (v/v) of water-miscible solvents (polar solvents), with the exception of acetone, esterase activity was either increased or retained stable. Addition of 50% (v/v) DMSO did not affect esterase activity, while addition of 50% (v/v) methanol, ethanol, 2-propanol, 1-butanol, or acetonitrile decreased enzymatic activity dramatically (Table 1).

**Table 1:** Effect of various organic solvents on EstCS2 activity

Compound	Remaining activity (%) at concentration (%) of		
	
	10	20	50
Control	100	100	100
Methanol	119.9	101.4	12.6
Ethanol	117.3	138.2	8.7
2-propanol	132.5	131.3	4.7
1-butanol	99.3	98.2	2.4
Acetonitrile	118.3	92.1	4.4
DMSO	125.9	128.2	97.4
Acetone	44.8	15.6	3.3
DMF	110.3	115.1	50.9

### Hydrolysis of tertiary alcohol esters and ketoprofen ethyl ester by EstCS2

**Figure 5:**
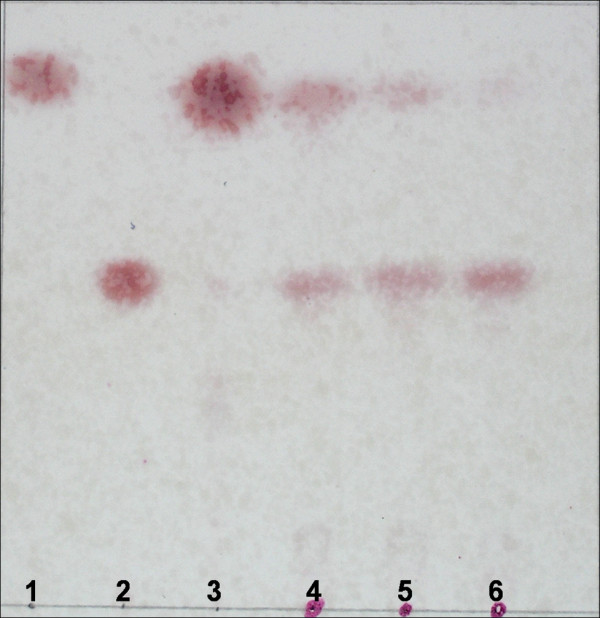
**TLC analysis of enzymatic conversion of racemic linalyl acetate to linalool**. The reaction was initiated by enzyme addition. lane 1, linalyl acetate ester; lane 2, linalool; lane 3, no enzyme added; lane 4, reaction time of 1h; lane 5 reaction time of 5 h; lane 6, reaction time of 10 h.

The ability of EstCS2 to hydrolyze esters of tertiary alcohols (TAs) was examined using linalyl acetate as a substrate. Thin-layer chromatography (TLC) analysis showed that EstCS2 converts linalyl acetate to linalool (Figure 5). The ability of EstCS2 to cleave sterically hindered esters of tertiary alcohol is remarkable. Enantioselectivity of EstCS2 was determined using racemic (R/S)-ketoprofen ethyl ester as a substrate. The time-dependent and substrate concentration-dependent enzymatic assay revealed that EstCS2 simultaneously hydrolyzes (R)-ketoprofen ethyl ester and (S)-ketoprofen ethyl ester, with a modest preference toward (R)-ketoprofen ethyl ester (ee_p _= -0.07%; ee%: enantiomeric excess).

## Discussion

We isolated a gene (*estCS2*) belonging to family VII from a metagenome library constructed from compost. Amino acid sequence analysis of EstCS2 exhibited the conserved sequence motifs of esterase/lipase, GXSXG, and putative catalytic triad composed of Ser_245_, Glu_363_, His_466_. EstCS2 preferred short-chain *p-*nitrophenyl esters as substrate, and it has optimal activity at pH 9.0 and 55°C. The enzyme is stable up to 60°C. This suggests that EstCS2 is an esterase which originates, most likely, from a thermostable microorganism in compost soil.

EstCS2 formed a clear zone on an indicator plate containing poly DEGA, which is one of components of polyester polyurethane. Therefore, EstCS2 seems to possess the ability to hydrolyze polyester polyurethane, although further enzymatic studies of polyurethane degradation are needed. The characterization of the polyurethanase is important for future research aimed at the development of new bioremediation techniques [21].

TAs and their esters are useful building blocks in chemistry, and they are regarded as important compounds from natural products to pharmaceuticals [22]. TLC analysis indicated that EstCS2 hydrolyzes esters of tertiary alcohol, such as linalyl acetate. Extensive amino acid sequence analysis showed that EstCS2 has a GGG(A)X motif, composed of G_160_GAF sequence. Lipolytic enzymes that possess a GGG(A)X motif at an oxyanion hole near the active site are active towards TAs, while most lipolytic enzymes with the more common GX motif cannot convert tertiary alcohol esters into TAs [22-24]. EstCS2 can also hydrolyze (R/S)-ketoprofen ethyl ester, with greater enantioselectivity for the (R)-enantiomer. In pharmaceutical applications, ketoprofen is one of the non-steroidal anti-inflammatory drugs (NSAID) with analgesic, antipyretic, and antiinflammatory effects and is known to inhibit the production of prostaglandins. Specifically, (S)-ketoprofen is a main compound associated with these effects, while the (R)-ketoprofen has unwanted side effects. Therefore, in order to use EstCS2 for the production of (S)-ketoprofen, (S)-specific enantioselectivity of EstCS2 would need to be improved through a directed evolutionary method, or structure-based rational design [25].

EstCS2 is stable in organic solvents. The discovery of natural enzymes that are stable in organic solvents is essential for their use in the chemical industry, but their discovery should also be accompanied with the development of biological, physical, and chemical methods [26].

In summary, a novel thermostable esterase, EstCS2, which belongs to family VII, was isolated from a compost metagenome library. EstCS2 has high stability in organic solvents; it can degrade polyurethane and hydrolyze ketoprofen ethyl ester and tertiary alcohol ester. These are very useful characteristics for biotechnological processes. This study demonstrates that the metagenomic approach is a useful technique to discover novel enzymes with potential for industrial applications.

## Materials and methods

### Screening of the esterase gene from a compost metagenomic fosmid library

Metagenomic DNA from compost soil was extracted as previously described [27], and it was used to construct a metagenomic library using the CopyControl™ fosmid library production kit (Epicentre, USA), according to the manufacturer's instructions. Activity-based esterase/lipase screening was performed to identify recombinant clones with lipase activity. The transformants were spread on Luria-Bertani (LB) agar plates containing chloramphenicol (25 μg/mL), 1% (v/v) tributyrin (C4) as a substrate, and 0.5% (w/v) gum arabic. Active colonies producing clear halos on the plates were selected, and the lipolytic genes were sequenced from the fosmids of active colonies by a random shotgun sequencing method. The open reading frame (ORF) of the esterase was compared with reference sequences that were retrieved from protein and nucleotide databases on the Entrez server at the NCBI (http://www.ncbi.nlm.nih.gov/sites/entrez/). Sequence similarity searches were performed using the BLAST 2.0 program, and multiple alignments were conducted with highly similar sequences with the CLUSTAL W program. A phylogenetic tree was constructed with the MEGALIGN program (DNA STAR Inc., USA).

### Cloning and overexpression of the *estCS2 *gene

The putative esterase gene was amplified by PCR with 2 primers (5'-GGGCATATGATGCGAGCCGAGTTGC-3' and 5'-GCGCTCGAGTGATTTTTGGGGATCT-3'; the underlined sequences indicate the *Nde*I and *Xho*I recognition sites, respectively). PCR products were digested with *Nde*I and *Xho*I, and then, they were ligated into the pET-22b(+) vector (Novagen, Germany) that had been digested with the same restriction enzymes. The recombinant DNA was transformed into *E. coli *BL21(DE3).

Transformants were cultured in LB liquid medium containing ampicillin (100 μg/mL) at 37°C. When the optical density of the culture reached an absorbance of around 0.6 at 600 nm, 0.5 mM isopropyl-β-d-thiogalactopyranoside (IPTG) was added for the induction of protein expression, and the transformants were incubated for additional 18 h at 21°C. The cells were harvested by centrifugation at 7,000 rpm at 4°C for 10 min and then resuspended in binding buffer (50 mM Tris-HCl at pH 8.0, containing 300 mM NaCl). The resuspended cells were disrupted by sonication, and the crude cell lysate was centrifuged at 16,000 rpm for 30 min; the supernatant, which constitutes the cell extract, was withdrawn. The cell extract was applied to a column containing Ni-NTA resin (QIAGEN GmbH, Germany). After washing with 25 column volumes of the binding buffer containing 2 mM imidazole, bound proteins were eluted with binding buffer containing 250 mM imidazole. Eluted fractions were concentrated by centrifugation at 500 × *g *at 4°C. Concentrated proteins were loaded onto a gel filtration column, Superdex 200 10/300 GL (GE Healthcare, USA), equilibrated with 50 mM Tris-HCl buffer (pH 8.0) containing 300 mM NaCl, and separation was conducted at a flow rate of 0.5 mL/min on a BioLogic DuoFlow Chromatography System (Bio-Rad Laboratories, USA). The resulting protein fractions were analyzed by SDS-PAGE in 10% polyacrylamide gels.

### N-terminal amino acid sequence analysis

N-terminal amino acid sequence analysis was performed on proteins that had been separated by SDS-PAGE and electrotransferred to PVDF membrane (Pro Blott; Applied Biosystems, USA). The gel was electroblotted at 50mA, for 60 min using freshly prepared 10 mM 3-(cyclohexylamino)-1-propanesulfonic acid (CAPS) buffer at pH 10 and 10% (v/v) methanol (HPLC grade) in distilled water as a blotting buffer. Subsequently, the gel was stained with Coomassie brilliant blue to confirm effective transfer. After blotting, selected protein bands were cut from the membrane, and N-terminal sequence analysis was carried out using an automatic protein sequencer (Applied Biosystems, USA).

### Plate assay for degradation of polyurethane and triglyceride

Activity of the EstCS2, to degrade polyurethane as substrate, was tested on an indicator plate. The polyurethane esterase indicator plate was prepared with a sonicated emulsion of 0.5% poly DEGA in 20 mM Tris-HCl at pH 8.0 [16]. The lipolytic activity indicator plate contained emulsified tributyrin. A crude cell extract of *E.coli *BL21(DE3)/pET22b, and purified EstCS2, were transferred into holes in the plates, and each plate was incubated for 5 h at ambient temperature.

### Characterization of EstCS2

Substrate preference toward *p*-nitrophenyl esters (C2-C18) was determined enzymatically by measuring the amount of *p*-nitrophenol released by hydrolysis. Absorbance was continuously measured at 405 nm for 4 min, using a DU800 spectrophotometer (Beckman, USA). As a standard assay solution, a mixture (1:4:95, by vol) of 10 mM *p*-nitrophenyl caprylate (C8), ethanol, and 100 mM GTA buffer was used. One unit of enzyme activity was defined as the amount of enzyme needed to release 1 μmol of *p*-nitrophenol per min at 25°C. Protein concentration was determined according to the method of Bradford (Bio-Rad Protein Assay), using bovine serum albumin as the standard. Substrate specificities toward various triacylglycerols were measured by titrating free fatty acid released by substrate hydrolysis. Substrates were emulsified in a solution containing 10 mM NaCl, 1 mM CaCl_2_, and 0.5% (w/v) gum arabic solution. The substrate emulsion (30 mL) was adjusted to pH 8.0 with 10 mM NaOH, and the reaction was initiated by adding purified enzyme. The pH of the reaction was recorded by a pH-stat (842 Titrando; Metrohm, USA), equipped with thermostat and stirrer, at 25°C for 6 min. One unit of esterase activity was defined as the amount of enzyme that liberates 1 μmol of fatty acid per min.

The optimum pH for enzyme activity was determined at 25°C in 100 mM GTA buffer, in the pH range 4.0-11.0. pH stability of the esterase was determined by incubating the enzyme in 100 mM GTA buffer (from pH 4-11) for 16 h at 4°C, and residual activity was measured at 25°C. To determine the optimum reaction temperature, the reaction mixture was incubated for 10 min at various temperatures (10-80°C). Thermostability of the esterase was determined by preincubation for 1 h, over a temperature range of 10-80°C, in 100 mM GTA buffer (pH 9.0); subsequently, residual activity was measured at 25°C.

To measure the effects of detergents on the esterase activity, aliquots of purified enzyme, mixed with various detergents and metal ions, were incubated in 100 mM GTA buffer (pH 9.0) at 30°C for 1 h. After incubation, residual activity was measured under standard assay conditions.

The effect of several inhibitors (at 1 mM, 5 mM, and 10 mM), such as PMSF, dithiothreitol (DTT), ethylenediaminetetraacetic acid (EDTA), and 2-mercaptoethanol were tested by incubation with the enzyme for 1 h at 30°C. The stability of the enzyme in the presence of organic solvents was tested with 10%, 20%, and 50% (w/v) of methanol, ethanol, 2-propanol, 1-butanol, acetonitrile, dimethyl sulfoxide (DMSO), acetone, and dimethylformamide (DMF) by incubation of the enzyme for 1 h at 30°C in various organic solvent dilutions.

### Hydrolysis of linalyl acetate and ketoprofen ethyl ether by EstCS2

The reaction mixture, containing *rac*-linalyl acetate (15 mg/mL) and the purified protein (10 mg/mL) in 50 mM Tris-HCl (pH 8.0), was agitated on a rotary shaker (200 rpm) at ambient temperature. The reaction was stopped by the addition of 4 volumes of ethanol, and insoluble material was removed by centrifugation. Samples were periodically withdrawn and analyzed by TLC using Merck silica gel 60 F_254_, and petroleum ether:ethyl acetate (5:1) as an eluent. Compounds were visualized by spraying with a solution of 5 g vanillin/L concentrated H_2_SO_4 _and subsequently heating.

(R/S)-Ketoprofen alkyl ester was synthesized by a general method for esterification. (R/S)-Ketoprofen (12 g) was solubilized in 100 mL of 100% ethanol in a round-bottom flask. H_2_SO_4 _was then added as a catalyst for esterification. The mixture was refluxed for 6 h at 70°C, with stirring, for the synthesis of ketoprofen ethyl ester. Sodium acetate (0.5 g) was added to quench the catalyst, the residual alcohol was removed by vacuum evaporation, and the esterified material was washed 3 times with aqueous 1 M NaHCO_3 _to remove unreacted ketoprofen, sulfuric acid, and ethanol [28]. The hydrolytic activity, using the (R/S)-ketoprofen ethyl ester as substrate, was determined at 30°C for 24 h with the purified enzyme (6 mg/mL) in a reaction mixture containing 50 mM Tris-HCl (pH 8.0). The reaction was stopped by the addition of 1 volume of ethanol, and insoluble material was removed by centrifugation. The resulting solution was then analyzed for conversion yield and enantioselectivity by high performance liquid chromatography (HPLC). The concentrations of (R/S)-ketoprofen ethyl ester, (R)-ketoprofen and (S)-ketoprofen were determined using an HPLC system (Hitachi, Japan). The column used was a chiral compound analytical column (Chirex Phase 3005; Phenomenex). Methanol containing 30 mM ammonium acetate was used as the mobile phase. The column was run at a constant flow rate (1.5 mL/min), and the eluent was monitored spectrophotometrically at 254 nm. One unit of enzyme activity was defined as the amount of enzyme producing 1 μmol of ketoprofen per min under the specified conditions.

### Nucleotide sequence accession number

The nucleotide sequence data of the esterase gene (*estCS2*) has been deposited in Gen Bank, under the accession number GU256649.

## Competing interests

The authors declare that they have no competing interests.

## Authors' contributions

CHK conducted the design of experiments, carried out the study, and drafted the manuscript. KHO and MHL assisted in the analysis of data and reviewed the manuscript. TKO participated in the design of experiments and helped to draft the manuscript. JHY and BHK conceived this study and are responsible for the design and drafting of the manuscript. All authors read and approved the final manuscript.
